# Ecology and geography of Cache Valley virus assessed using ecological niche modeling

**DOI:** 10.1186/s13071-024-06344-z

**Published:** 2024-06-26

**Authors:** John A. Muller, Krisangel López, Luis E. Escobar, Albert J. Auguste

**Affiliations:** 1 Department of Entomology, College of Agriculture and Life Sciences, Fralin Life Science Institute, Virginia Polytechnic Institute and State University, Blacksburg, VA 24061 USA; 2 Department of Fish and Wildlife Conservation, Virginia Polytechnic Institute and State University, Blacksburg, VA 24061 USA; 3 Center for Emerging, Zoonotic, and Arthropod-Borne Pathogens, Virginia Polytechnic Institute and State University, Blacksburg, VA 24061 USA

**Keywords:** Cache Valley virus, Ecological niche modeling, White-tailed deer, Mosquito, Vector

## Abstract

**Background:**

Cache Valley virus (CVV) is an understudied *Orthobunyavirus* with a high spillover transmission potential due to its wide geographical distribution and large number of associated hosts and vectors. Although CVV is known to be widely distributed throughout North America, no studies have explored its geography or employed computational methods to explore the mammal and mosquito species likely participating in the CVV sylvatic cycle.

**Methods:**

We used a literature review and online databases to compile locality data for CVV and its potential vectors and hosts. We linked location data points with climatic data via ecological niche modeling to estimate the geographical range of CVV and hotspots of transmission risk. We used background similarity tests to identify likely CVV mosquito vectors and mammal hosts to detect ecological signals from CVV sylvatic transmission.

**Results:**

CVV distribution maps revealed a widespread potential viral occurrence throughout North America. Ecological niche models identified areas with climate, vectors, and hosts suitable to maintain CVV transmission. Our background similarity tests identified *Aedes vexans*, *Culiseta inornata*, and *Culex tarsalis* as the most likely vectors and *Odocoileus virginianus* (white-tailed deer) as the most likely host sustaining sylvatic transmission.

**Conclusions:**

CVV has a continental-level, widespread transmission potential. Large areas of North America have suitable climate, vectors, and hosts for CVV emergence, establishment, and spread. We identified geographical hotspots that have no confirmed CVV reports to date and, in view of CVV misdiagnosis or underreporting, can guide future surveillance to specific localities and species.

**Graphical Abstract:**

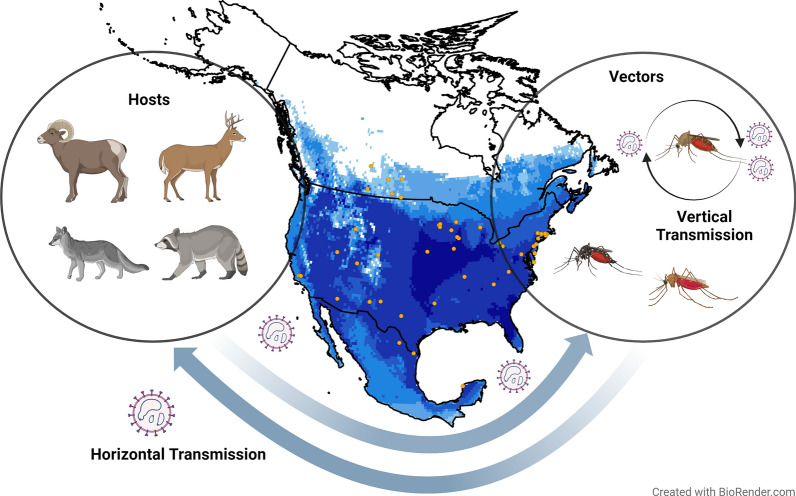

**Supplementary Information:**

The online version contains supplementary material available at 10.1186/s13071-024-06344-z.

## Background

Cache Valley virus (CVV) is an arthropod-borne virus in the genus *Orthobunyavirus* [1] that was first isolated in 1956 in Cache Valley, Utah (USA) [2]. Since its initial isolation, CVV has been found to be widely distributed throughout North America [1]. The virus is known to cause encephalitis and fatalities in humans, and spontaneous abortions and congenital abnormalities in ruminants [1, 3]. A total of seven human CVV infections have been reported, three of which were fatal [4–8]. Fetal macrocephaly and developmental delays in humans have been retroactively associated with CVV via cases of mothers who were infected during pregnancy [9], suggesting that the morbidity of the disease may be higher than current reports. There are no robust data quantifying CVV incidence among human populations.

Although little is known about CVV incidence and prevalence in humans, over the last 60 years, the impact of CVV on agriculture and livestock has not gone unnoticed. For example, CVV has been observed in a wide variety of livestock species, including sheep (*Ovis* sp.), goats (*Capra hircus*), cattle (*Bos taurus*), horses (*Equus caballus*), and swine (*Sus scrofa*), most of which are found throughout North America [3, 10–12]. Serosurveillance in livestock revealed 96.4%, 53.3%, and 58.9% prevalence in the eastern, central, and western United States, respectively [3, 13]. Human seroprevalence has been estimated at ~18% within the United States [1, 3]. In wildlife populations, CVV has shown seropositivity across taxa, from ruminants to carnivores to lagomorphs, with species including white-tailed deer (*Odocoileus virginianus*), mule deer (*O. hemionus*), elk (*Cervus elaphus*), swift foxes (*Vulpes velox*), kit foxes (*V. macrotus*), raccoons (*Procyon lotor*), cottontail rabbits (*Sylvilagus floridanus*), and jackrabbits (*Lepus californicus*) [10, 11, 14–17]. It is unknown which of these wildlife species are contributors to the distribution and sylvatic maintenance of CVV. In addition to CVV’s wide host range, the virus also has a large potential vector range. Mosquitoes from the genera *Aedes*, *Anopheles*, *Coquillettidia*, *Culex*, *Culiseta*, *Mansonia*, and *Psorophora* are reported as plausible CVV vectors [1, 12, 18]. The primary CVV vector, however, remains unknown. Nevertheless, although the primary vector and reservoir host species remain unknown, the life cycle of CVV is likely maintained both in a dual-host cycle between various mosquito vectors and mammalian hosts and within vector species through vertical transmission [10, 19].

Despite evidence of CVV incidence across North America and the broad list of host and vector species, the biogeography of CVV transmission risk remains unexplored. By creating ecological niche models for known CVV vectors, wildlife hosts, and susceptible hosts, and coupling these models with CVV reported cases, we created a map of CVV transmission risk and identified the most likely CVV hotspots, primary host, and primary vectors based on niche theory.

## Methods

### Data acquisition

CVV location data were compiled from various sources including an extensive literature search from Google Scholar and PubMed using the search term “Cache Valley virus,” as well as metadata from pathogen repositories including the Arbovirus Reference Collection at the Centers for Disease Control and Prevention, GenBank, and the World Reference Center for Emerging Viruses and Arboviruses (WRCEVA) at the University of Texas Medical Branch, Galveston, Texas (Additional file 1: Table S2). The literature search was conducted until December 2022, and the query period encompassed 1959–2022. During literature reviews, data were gathered from papers that reported either positive mosquito samples or seropositive wildlife or captive animals. Only locations with specific site data were used (e.g., for uncertainty greater than 30 km, the record was discarded). Our literature review found 51 locations for CVV that had sufficiently high spatial specificity to be used (Additional file 1: Table S2). After rarefaction of points, we ended up with 47 locations to be used in Maxent modeling.

We compiled occurrence records for 41 species of mosquito vectors that are associated with CVV transmission, i.e., in which CVV was isolated from the species or shown to be a competent vector in laboratory settings (Additional file 1: Table S1). We found 11 wildlife species considered potential CVV hosts, namely, those with the presence of CVV infection or neutralizing antibodies (Additional file 1: Table S1). Vector and host occurrence data were obtained from the Global Biodiversity Information Facility (GBIF) [20] and curated for uncertainty (i.e., locations with uncertainty greater than 10 km, occurred outside North America, or identified as an unsuitable habitat, e.g., open ocean). Occurrences were spatially rarefied by removing autocorrelated points that were within the same pixel [21], and only species with > 15 occurrences were modeled.

Nine environmental layers available from Chelsa climate variables at partial resolution of ~1 km, resampled to a resolution of 30 km for reduced processing power, were used to estimate suitable climate for the species [22]. We removed four interactive variables from the original set of 19 because of discontinuities [23]. Climatic variables were tested for autocorrelation via Pearson’s correlation coefficient in the ENMTools R package, and an additional six redundant layers (*r* > 0.9) were removed [24]. The final variables selected for the model calibration and their description can be found in the supplementary materials (Additional file 1: Table S3).

### Maxent modeling

Distribution maps were constructed using Maxent v3.4.1 [25] in the ENMTools R package [24]. Maxent is a program used to model species distributions and is a widely employed presence–background method [26]. Even though Maxent cannot estimate relative abundance and does not model occurrence probability [26], it accurately estimates suitable environments mirroring the environment occupied by the organism. Maxent uses presence–background data, which allows us to model species with limited occurrence data. During model calibration, we tested 35 candidate models that were a combination of seven regularization multipliers (0.5, 1, 1.5, 2, 3, 4, 5) and five feature combinations (linear, linear+quadratic, linear+quadratic+hinge, hinge, linear+quadratic+hinge+product+threshold). There were five replicates for each species based on *k*-fold cross-validation [21, 27, 28]. Omission rates and area under the curve (AUC) were used to evaluate models, and the model with the lowest omission rate was selected to prioritize prediction performance of independent data.

Maxent models of vectors, hosts, and CVV were converted to binary using a 10% training presence threshold [28]. Use of this threshold results in a more conservative estimation of suitable distribution, and it is also less affected by extremes that can occur in small datasets [29]. Once all of the thresholded wildlife host and mosquito vector models were converted into binary models, we developed a model ensemble by summing the binary rasters using the cell statistics tool in ArcMap to create a map of potential species richness.

### Background similarity test

Niche similarity of vector and host models was compared to the CVV model using background similarity tests, resulting in a Schoener’s *D* value [24]. The Schoener’s *D* value, which ranges from 0 to 1, shows the similarity between the geographical predictions of two niches, where a higher value indicates a higher degree of similarity. The background similarity test compared the Schoener’s *D* values obtained in the observed comparison models generated against samples drawn randomly from the study area of one species against the estimated range of another species in a series of permutations (*n* = 100) [30]. Permutations were used to create a null distribution of potential *D* values, and from this null distribution a 95% critical value was derived from the lower tail of the distribution.* D* values that fell below the critical value were interpreted as having a niche more dissimilar to CVV than that due to chance (*P* < 0.05). Study areas were generated by creating buffers of 500 km around each point location. A final risk model was generated by combining the binary models of the top 10 host and vector species with the highest niche similarity to the CVV’s niche model.

## Results

### Distribution and richness models

**Fig. 1:**
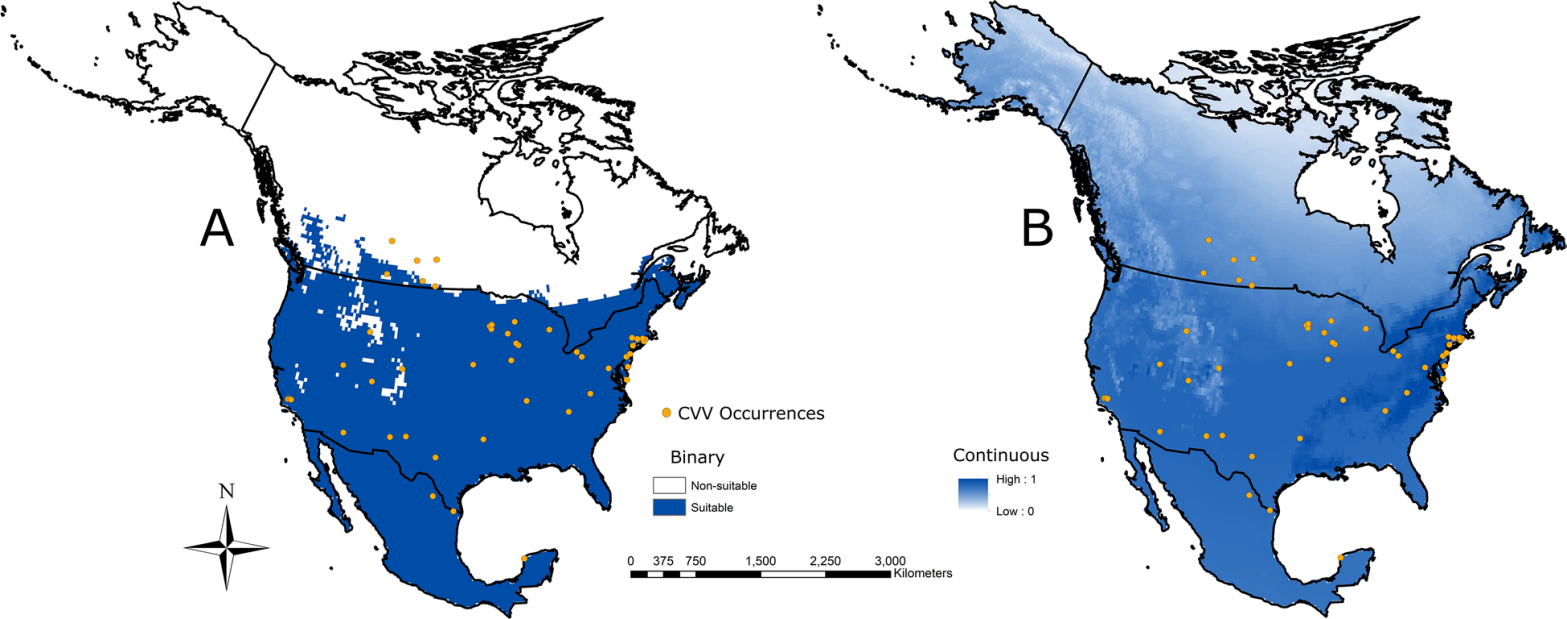
Ecological niche models for Cache Valley virus. **A** Binary model shows the area that is modeled suitable in blue and unsuitable in white. **B** Continuous model shows areas that are darker as the more suitable areas while areas that are lighter are less suitable. The AUC for the model was 0.694. Orange dots are locations in which CVV was detected. Binary models were constructed using a 10% training presence threshold in the Maxent program, meaning that 10% of points that occur in the least suitable environments are deemed not representative of the ideal conditions for the organism modeled, and are given an unsuitable score

The greatest concentration of points occurred in the Northeast along the coast, and in the Midwest states, while most of Mexico and Canada had no points (Fig. 1). The binary CVV ecological niche model revealed a widespread CVV potential distribution throughout North America, including Mexico, most of the United States, and southern regions of Canada (Fig. 1A). The continuous CVV niche model showed the areas with the highest suitability as east of the Appalachian Mountains, and parts of the southeastern USA including parts of Louisiana, East Texas, and Arkansas (Fig. 1B).

**Fig. 2:**
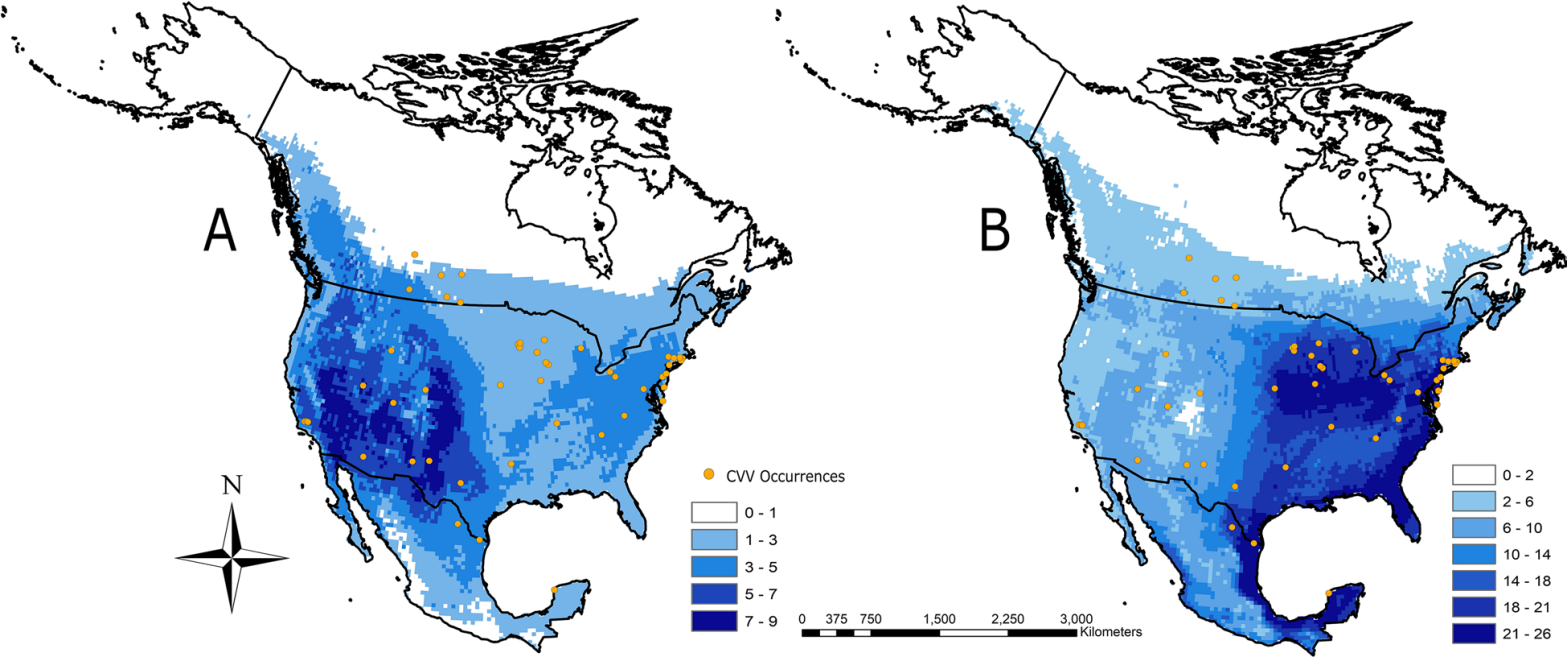
Species richness models (i.e., predicted number of species within each pixel) for the potential hosts (**A**) and vectors (**B**) for CVV in North America. For both models, darker colors represent increasing numbers of species. Both models were constructed by adding the binary models of all of the hosts and/or vectors in each group together. Binary models were constructed using a 10% training presence threshold in the Maxent program, meaning that 10% of points that occurred in the least suitable areas are deemed not representative of the ideal conditions for the organism modeled, and are given an unsuitable score. Orange dots are locations in which CVV was detected

After filtering and removing species with insufficient data, a total of 36 out of the 41 suspected CVV vector species and 11 host species were used to create the models (Table 1). The AUC for the individual species models ranged from 0.614 to 0.921 but averaged 0.774 for hosts and 0.772 for vectors (Table 1). Ecological niche models of mosquitoes and wildlife show large areas of North America as having substantial numbers of potential vectors and hosts. A cluster of estimated richness of 7–9 hosts was found in the southwestern USA (Fig. 2A). Vectors, however, showed high levels of expected species richness (21–26 species) clustered in multiple locations in eastern North America—specifically, along the Gulf coast of Mexico into the Yucatan Peninsula, the US Midwest states of Iowa, Illinois, and Michigan, and along the US East Coast from Florida and Georgia, north to areas of New Jersey and Maryland (Fig. 2B). We observed better agreement between the cluster of CVV cases with the richness of vectors than with the richness of hosts.

**Table 1:** Full results from the background test comparison to the Cache Valley virus niche model

Species	Common name	Host/vector	Schoener’s *D*	95% critical value	Significantly dissimilar	Included in final risk model	AUC
*Aedes vexans*	Inland floodwater mosquito	Vector	0.665	0.504	No	Yes	0.772
*Culiseta inornata*	Winter marsh mosquito	Vector	0.633	0.535	No	Yes	0.845
*Odocoileus virginianus*	White-tailed deer	Host	0.609	0.55	No	Yes	0.739
*Procyon lotor*	Northern racoon	Host	0.556	0.559	Yes	No	0.682
*Culex tarsalis*	Western encephalitis mosquito	Vector	0.541	0.525	No	Yes	0.794
*Aedes sticticus*	Floodwater mosquito	Vector	0.539	0.431	No	Yes	0.716
*Coquillettidia perturbans*	Cattail mosquito	Vector	0.508	0.522	Yes	No	0.782
*Aedes canadensis*	Woodland pool mosquito	Vector	0.501	0.352	No	Yes	0.775
*Cervus elaphus*	Elk	Host	0.456	0.527	Yes	No	0.859
*Culex pipiens*	Common house mosquito	Vector	0.451	0.442	No	Yes	0.738
*Odocoileus hemionus*	Mule deer	Host	0.438	0.473	Yes	No	0.779
*Culex restuans*	Northern house mosquito	Vector	0.432	0.497	Yes	No	0.774
*Sylvilagus floridianus*	Eastern cottontail	Host	0.431	0.537	Yes	No	0.714
*Aedes trivittatus*	Floodwater nuisance mosquito	Vector	0.402	0.433	Yes	No	0.756
*Aedes fitchii*	Woodland mosquito	Vector	0.397	0.269	No	Yes	0.643
*Anopheles freeborni*	Western malaria mosquito	Vector	0.386	0.312	No	Yes	0.767
*Culiseta melanura*	Black-tailed mosquito	Vector	0.381	0.429	Yes	No	0.894
*Anopheles punctipennis*	Woodland malaria mosquito	Vector	0.38	0.48	Yes	No	0.816
*Marmota monax*	Groundhog	Host	0.378	0.393	Yes	No	0.839
*Aedes cinereus*	Woodland mosquito	Vector	0.372	0.286	No	Yes	0.764
*Aedes sollicitans*	Eastern saltmarsh mosquito	Vector	0.367	0.344	No	No	0.775
*Anopheles walkeri*	Malaria mosquito	Vector	0.353	0.351	No	No	0.740
*Lepus californicus*	Black-tailed jackrabbit	Host	0.311	0.438	Yes	No	0.826
*Anopheles quadrimaculatus*	Common malaria mosquito	Vector	0.308	0.398	Yes	No	0.747
*Aedes triseriatus*	Eastern tree hole mosquito	Vector	0.298	0.301	Yes	No	0.714
*Aedes japonicus*	Asian bush mosquito	Vector	0.297	0.369	Yes	No	0.797
*Culex salinarius*	Unbanded saltmarsh mosquito	Vector	0.288	0.285	No	No	0.614
*Ovis canadensis*	Bighorn sheep	Host	0.283	0.4	Yes	No	0.786
*Anopheles crucians*	Malaria mosquito	Vector	0.277	0.277	No	No	0.804
*Psorophora ferox*	White-footed woods mosquito	Vector	0.25	0.275	Yes	No	0.776
*Aedes aegypti*	Yellow fever mosquito	Vector	0.237	0.408	Yes	No	0.735
*Aedes albopictus*	Asian tiger mosquito	Vector	0.235	0.383	Yes	No	0.793
*Aedes communis*	The pollinating mosquito	Vector	0.233	0.22	No	No	0.713
*Culex quinquefasciatus*	Southern house mosquito	Vector	0.213	0.294	Yes	No	0.728
*Aedes stimulans*	Woodland mosquito	Vector	0.203	0.341	Yes	No	0.686
*Aedes scapularis*	Mosquito	Vector	0.182	0.108	No	No	0.802
*Culex pilosus*	Floodwater mosquito	Vector	0.181	0.14	No	No	0.728
*Aedes cantator*	Brown saltmarsh mosquito	Vector	0.157	0.197	Yes	No	0.921
*Aedes taeniorhynchus*	Black saltmarsh mosquito	Vector	0.134	0.208	Yes	No	0.885
*Culex nigripalpus*	Florida SLE mosquito	Vector	0.121	0.142	Yes	No	0.815
*Mansonia titillans*	Freshwater mosquito	Vector	0.116	0.132	Yes	No	0.880
*Vulpes macrotis*	Kit fox	Host	0.103	0.223	Yes	No	0.773
*Aedes serratus*	Mosquito	Vector	0.099	0.119	Yes	No	0.780
*Anopheles albimanus*	Malaria mosquito	Vector	0.092	0.119	Yes	No	0.805
*Ovis dalli*	Dall sheep	Host	0.089	0.155	Yes	No	0.712
*Vulpes velox*	Swift fox	Host	0.08	0.29	Yes	No	0.815
*Culex corniger*	Mosquito	Vector	0.074	0.103	Yes	No	0.720

The 95% critical value is derived from the null distribution from the background test; if the *D* value is higher than the critical value, then the two are not significantly dissimilar. The area under the curve (AUC) is presented for model accuracy. Species are ranked with the highest Schoener’s *D* value shown first. SLE, St. Louis encephalitis

### Niche similarity

Sixteen mosquito species showed similar ecological niches to CVV (non-dissimilar *D* score via background similarity test). Specific mosquito species were classified as highly similar to the ecological niche of CVV occurrences, suggesting high ecological correspondence or likelihood of playing an important role in the maintenance of CVV transmission (Table 1). The three species with the highest niche similarity were *Aedes vexans*, *Culiseta inornata*, and *Culex tarsalis*, with niche similarity values of 0.665, 0.633, and 0.541, respectively (Table 1). Other vector species with high niche similarity *D* scores to CVV that were shown to be significantly different with background similarity tests included *Coquillettidia perturbans*, *Culex restuans*, and *Aedes trivittatus*, with *D* scores of 0.508, 0.432, and 0.402, respectively (Table 1).

The host species with the highest ecological similarity to CVV were *O. virginianus*, *P. lotor*, and *Cervus elaphus*, with *D* scores of 0.609, 0.556, and 0.456, respectively, although only *O. virginianus* had a non-significantly different niche when compared using the background similarity test (Table 1). Other potential hosts had very low niche similarity to CVV, such as *Vulpes* spp. (0.103 and 0.08 *D* scores) and *Ovis* spp. (0.283 and 0.089 D scores) (Table 1). Only *O. virginianus* and *P. lotor* occur in the eastern half of North America, where the majority of CVV locations have been found (Fig. 2A).

### Final risk model

**Fig. 3:**
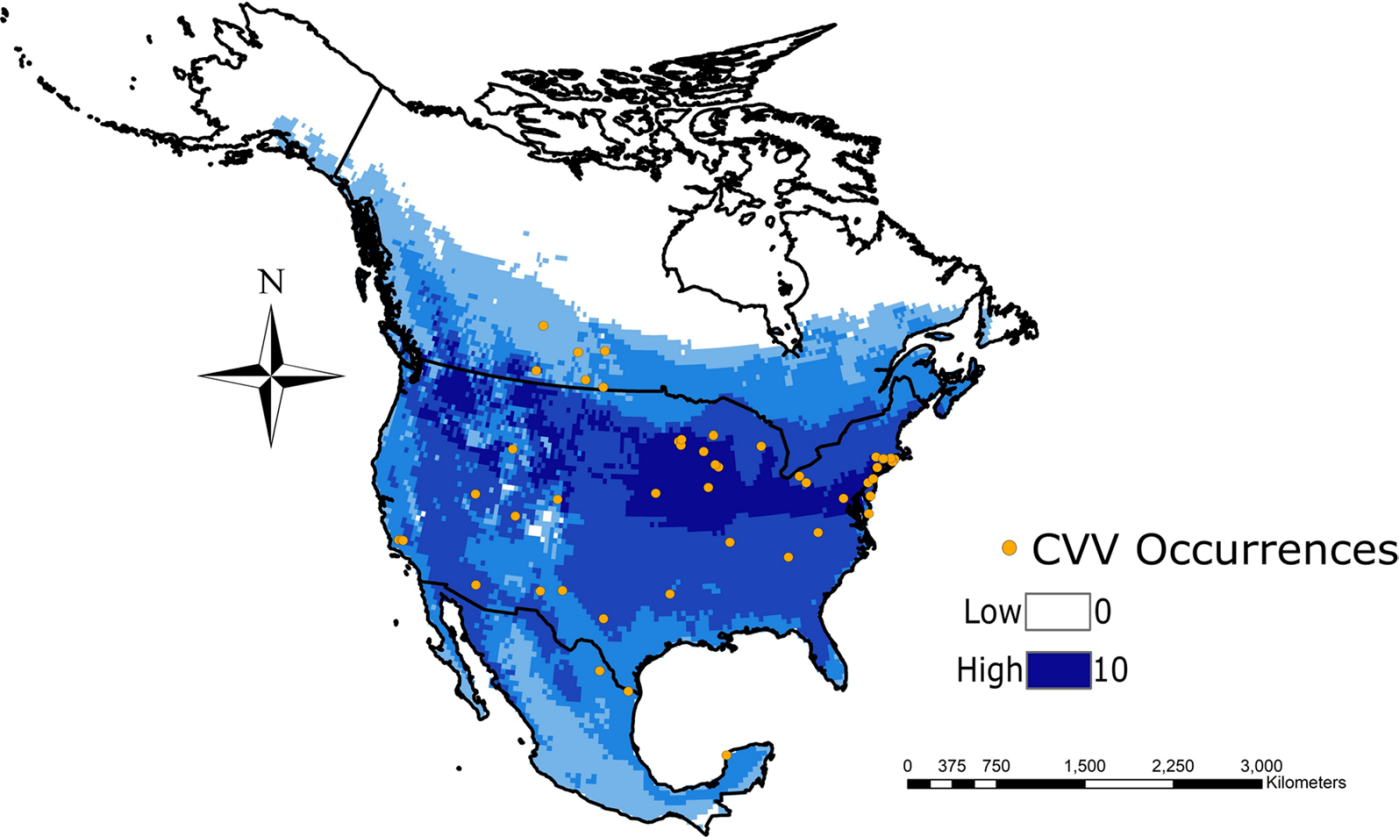
Final CVV risk model for CVV in North America. Darker colors represent increasing numbers of species. The model was constructed by adding together the binary models of all of the hosts and/or vectors in each group. Binary models were constructed using a 10% training presence threshold in program Maxent, meaning that 10% of points that occurred in the areas least suitable are deemed not representative of the ideal conditions for the organism modeled, and are given an unsuitable score. Orange dots are locations in which CVV was detected

The final CVV risk model included the top 10 species with the highest, significant niche similarity to CVV. This model ensemble included nine vector species and one host species (Table 1). The model shows that although the potential for CVV distribution is widespread across North America, there are areas with higher potential for transmission. Among the areas highlighted by the model as having high potential for CVV transmission are those along the eastern Great Plains, US Midwest, and northwestern USA on either side of the Rocky Mountains including parts of Washington, Montana, and Wyoming (Fig. 3). Southern Canada within the Great Plains provinces and northern Mexico, particularly along the Gulf coast and northern Baja, were also highlighted as potential areas.

## Discussion

To properly prepare for potential CVV spillover transmission events from wildlife to humans and livestock, and to anticipate CVV outbreaks, a deeper understanding of risk areas and species able to sustain transmission is critical. Our models showed large areas of North America that are suitable for CVV transmission in its sylvatic cycle. Within those areas there are large swaths of regions that have no reports of CVV occurrence (e.g., Oklahoma, Washington, Montana, and Kansas in the central and northwestern USA, and southern Mexico).

Although we see an incongruence between our estimated CVV distribution and known CVV case reports, we believe this is due to underreporting or misdiagnosis. A recent outbreak of CVV in the US state of Arkansas highlights the likelihood of underreporting [31]. Arkansas, which is in the southern USA, is an area that our final model highlighted as having fairly high potential for sylvatic CVV maintenance but presented no previous reports in the region, thus giving validity to our model. Locations outside North America with reported CVV occurrences, such as Jamaica, Trinidad and Tobago, and Argentina, likely reflect the reclassified Maguari virus [32]. Maguari virus is antigenically related and significantly cross-reacts with CVV serologically [32]. Arbovirus-associated encephalitis cases often go undiagnosed, as was evidenced by a retroactive surveillance study for bunyaviruses that showed high prevalence and a plausible link between CVV infection and macrocephaly in infants [9]. Additionally, there may be vector and host species assessed in these models that are incompetent for CVV transmission and thus do not contribute to CVV maintenance. Further vector and host competency studies are needed so we can narrow the list of species which may influence the model. Further surveillance and increased testing are needed across North America to thoroughly assess CVV distribution and prevalence.

By learning which species are the likeliest sylvatic contributors to CVV distribution, we can more efficiently select which species to monitor in the future. There were 30 species of vectors and hosts that did not present significant similarity with CVV niches, suggesting they are unlikely to have important roles in CVV maintenance and distribution. Therefore, previous reports may represent accidental infections without necessary maintenance of transmission.

*Aedes vexans,* the species with the most similar niche to CVV, was shown to be a mildly effective vector in laboratory settings, implying a limited role in sylvatic cycles [33]. The second most similar species to CVV is *Culiseta inornata*, which has been shown to be a competent vector for CVV and efficiently transmits the virus both horizontally and vertically [34]. Other species with high niche similarity such as *Culex pipiens* have been shown to be incompetent vectors in laboratory settings [35] or have not yet been tested. Few vector species have been experimentally tested for vertical transmission of CVV, despite the importance of vertical transmission in maintenance and continuous local transmission of CVV in a given region. To determine the most plausible primary vectors for CVV, more vector competence studies are urgently needed, especially for the species predicted here as ecologically similar to CVV in occurrence (i.e., *Aedes canadensis, Ae. fitchii, Ae. cinereus*, and *Anopheles freeborni*). Identifying those species that are suitable vectors for CVV and those capable of vertical transmission will also help inform more accurate risk models by excluding species incapable of CVV transmission. Once these additional competency studies have been conducted and we have a better understanding of the natural life cycle of CVV, finer-scale niche modeling with the addition of non-climatic variables (e.g., topography and vegetation cover), as well as with the smaller group of more important sylvatic contributors, would be a worthy future study to obtain a more precise risk map of CVV distribution.

It is likely that the most prominent CVV vector species vary geographically. For example, in the northeastern USA, *Anopheles* spp. are implicated as the primary vectors [18, 36], but in the western USA, *Anopheles* spp. are unlikely to drive CVV circulation given their limited distribution in that region [20, 37]. Furthermore, in the northeastern USA, CVV has recently undergone a lineage displacement, with lineage 2 becoming the predominant lineage in the region, and this was shown to be driven in part by the increased competency in *Anopheles* spp. with lineage 2 strains [36]. Further surveillance is needed in the western USA to determine whether lineage 1 is still predominant.

Interestingly, a dissimilarity was found between host and vector richness models, where vectors have much higher richness in the eastern USA, and the host richness is much higher in the western USA. This may be a reflection of the fact that mosquito diversity is higher in the eastern USA than in the West [37]. Nonetheless, this does not preclude the possibility that CVV circulation and distribution is being driven predominantly by hosts in the western USA and by vectors in the east. If CVV abundance is more closely tied to mosquito diversity and abundance, this could mean that the eastern half of North America is more at risk of CVV infection and emergence. Future surveillance studies are needed to address whether species richness impacts the distribution or abundance of CVV.

*Odocoileus virginianus* demonstrated the highest niche overlap with CVV among wildlife hosts, supporting previous studies which showed that experimentally infected *O. virginianus* do become viremic and at high enough titers to transmit CVV [10]. Multiple wildlife species including *Ovis* spp. and *Vulpes* spp. showed very little overlap with CVV and therefore may play a more limited role in the distribution of CVV, but their role in maintaining viral circulation is not discarded. Given that many domestic livestock species (e.g., cattle, horses, sheep, and goats) are CVV hosts, livestock likely play a role in the overall distribution and maintenance of CVV. Even though there are multiple domestic livestock species that could influence CVV distribution, the influence of anthropogenic factors in modeling livestock distribution is especially challenging [38] in light of the congruency issues with various existing livestock datasets. We therefore decided to focus on natural sylvatic transmission cycles and removed livestock modeling from our analysis.

## Conclusions

Given the diversity of vectors and hosts and the widespread distribution of CVV, we conclude that CVV circulation is primed for potential outbreaks. The broad risk estimate for CVV transmission suggests future outbreaks in livestock in the areas predicted by our models. CVV circulation in wildlife and its emergence in livestock should be considered an early warning for CVV spillover to humans. As such, CVV is an ideal important arboviral pathogen model for One Health research and management, and the validation of our predictions empirically warrants further study. Increased surveillance is urgently needed in the hotspots of transmission risk predicted here in order to better understand CVV prevalence in natural and disturbed ecosystems. A more detailed understanding of CVV ecology can inform effective intervention strategies needed to prevent CVV emergence in humans and animals.

### Supplementary Information

Additional file 1.

## Data Availability

All data is publicly available from online sources such as GenBank, Arboviral Reference Collection at the Centers for Disease Control and Prevention, Global Biodiversity Information Facility, and literature review.
