# Supplementary material for: Ecology and geography of Cache Valley virus assessed using ecological niche modeling
Source: Parasit Vectors. 2024 Jun 26;17:270. doi: 10.1186/s13071-024-06344-z (PMC11210180; doi:10.1186/s13071-024-06344-z)
Supplement: Supplementary file 1 — Additional file 1. [file 13071_2024_6344_MOESM1_ESM.docx]

Table S1

Citations used to during literature review for inclusion of each species of host and vector in the models. For many species there were greater than 3 citations but here we limit to 3. If a species was excluded, we comment why in the comments’ column.

| Species | Host/ Vector | Citation 1 | Citation 2 | Citation 3 | Comments |
| --- | --- | --- | --- | --- | --- |
| *Aedes aegypti* | Vector | Edwards, J. F., Higgs, S., & Beaty, B. J. (1998). Mosquito feeding-induced enhancement of Cache Valley virus (bunyaviridae) infection in mice. *Journal of Medical Entomology, 35*, 261-265. | Belle, E. A., King, S. D., Griffiths, B. B., Grant, L. S. (1980). Epidemiological investigation for arboviruses in Jamaica, West Indies. American *Journal of Tropical Medicine and Hygiene,* 29, 667-675. | Farfan-Ale, J., Lorono-Pino, M., Garcia-Rejon, J., Soto, V., Lin, M., Staley, M., . . . Blitvich, B. J. (2010). Detection of flaviviruses and orthobunyaviruses in mosquitoes in the Yucatan peninsula of Mexico in 2008. *Vector Borne Zoonotic Dis*, 10, 777-783. |  |
| *Aedes albopictus* | Vector | Andreadis, T. G., Armstrong, P. M., Anderson, J. F., & Main, A. J. (2014). Spatial-temporal analysis of Cache Valley virus (Bunyaviridae: Orthobunyavirus) infection in anopheline and culicine mosquitoes (diptera: Culicidae) in the northeastern United States, 1997-2012. *Vector-Borne and Zoonotic Diseases*, 14, 763-773. | Armstrong, P.M., Andreadis, T.G., Shepard, J.J., & Thomas, M.C . 2018. Northern range expansion of the Asian tiger mosquito (Aedes albopictus): Analysis of mosquito data from Connecticut, USA. PLoS *Neglected Tropical Diseases*. 11(5):e0005623. | [Armstrong, P.M., Anderson, J.F., Farajollahi, A., Healy, S.P., Unlu, I., Crepeau, T.N., Gaugler, R., Fonseca, D.M., & Andreadis, T.G. (2013). Isolations of Cache Valley virus from Aedes albopictus (Diptera: Culicidae) in New Jersey and evaluation of its role as a regional arbovirus vector. J](http://dx.doi.org/10.1603/ME13099)*[ournal of Medical Entomology](http://dx.doi.org/10.1603/ME13099)*[, 50, 1310-1314;](http://dx.doi.org/10.1603/ME13099) |  |
| *Aedes cantator* | Vector | Andreadis, T. G., Armstrong, P. M., Anderson, J. F., & Main, A. J. (2014). Spatial-temporal analysis of Cache Valley virus (Bunyaviridae: Orthobunyavirus) infection in anopheline and culicine mosquitoes (diptera: Culicidae) in the northeastern United States, 1997-2012. *Vector-Borne and Zoonotic Diseases*, 14, 763-773. | Buescher, E. L., Byrne, R. J., Clarke, G. C., Gould, D. J., Russell, P. K., Scheider, F. G., & Yuill, T. M. (1970). Cache Valley virus in the Delmarva peninsula. I. virologic and serologic evidence of infection. *American Journal of Tropical Medicine and Hygiene, 19*, 493-502. | Ngo, K. A., Maffei, J. G., Dupuis Ii, A. P., Kauffman, E. B., Backenson, P. B., & Kramer, L. D. (2006). Isolation of Bunyamwera serogroup viruses (bunyaviridae, orthobunyavirus) in New York state. *Journal of Medical Entomology*, 43, 1004-1009. |  |
| *Aedes canadensis* | Vector | Andreadis, T. G., Armstrong, P. M., Anderson, J. F., & Main, A. J. (2014). Spatial-temporal analysis of Cache Valley virus (Bunyaviridae: Orthobunyavirus) infection in anopheline and culicine mosquitoes (diptera: Culicidae) in the northeastern United States, 1997-2012. *Vector-Borne and Zoonotic Diseases, 14*, 763-773. | Buescher, E. L., Byrne, R. J., Clarke, G. C., Gould, D. J., Russell, P. K., Scheider, F. G., & Yuill, T. M. (1970). Cache Valley virus in the Delmarva peninsula. I. virologic and serologic evidence of infection. *American Journal of Tropical Medicine and Hygiene, 19*, 493-502. | Ngo, K. A., Maffei, J. G., Dupuis Ii, A. P., Kauffman, E. B., Backenson, P. B., & Kramer, L. D. (2006). Isolation of Bunyamwera serogroup viruses (bunyaviridae, orthobunyavirus) in New York state. *Journal of Medical Entomology*, 43, 1004-1009. |  |
| *Aedes cinereus* | Vector | Andreadis, T. G., Armstrong, P. M., Anderson, J. F., & Main, A. J. (2014). Spatial-temporal analysis of Cache Valley virus (Bunyaviridae: Orthobunyavirus) infection in anopheline and culicine mosquitoes (diptera: Culicidae) in the northeastern United States, 1997-2012. *Vector-Borne and Zoonotic Diseases, 14*, 763-773. | Anderson, J.F., Armstrong, P.M., Misencik, M.J., Bransfield, A.B., Andreadis, T.G., & Molaei, G. (2018). Seasonal distribution, blood-feeding habits, and viruses of mosquitoes in an open-faced quarry in Connecticut, 2010 and 2011. Journal of the *American Mosquito Control Association*, 34, 1–10. | Ngo, K. A., Maffei, J. G., Dupuis Ii, A. P., Kauffman, E. B., Backenson, P. B., & Kramer, L. D. (2006). Isolation of Bunyamwera serogroup viruses (bunyaviridae, orthobunyavirus) in New York state. *Journal of Medical Entomology*, 43, 1004-1009. |  |
| *Aedes communis* | Vector | Andreadis, T. G., Armstrong, P. M., Anderson, J. F., & Main, A. J. (2014). Spatial-temporal analysis of Cache Valley virus (Bunyaviridae: Orthobunyavirus) infection in anopheline and culicine mosquitoes (diptera: Culicidae) in the northeastern United States, 1997-2012. *Vector-Borne and Zoonotic Diseases, 14*, 763-773. | Ngo, K. A., Maffei, J. G., Dupuis Ii, A. P., Kauffman, E. B., Backenson, P. B., & Kramer, L. D. (2006). Isolation of Bunyamwera serogroup viruses (bunyaviridae, orthobunyavirus) in New York state. *Journal of Medical Entomology, 43*, 1004-1009. | Main, A. J. (1981). Arbovirus surveillance in Connecticut. IV. Bunyamwera group. Mosquito News, 41, 490-494. |  |
| *Aedes fitchii* | Vector | Andreadis, T. G., Armstrong, P. M., Anderson, J. F., & Main, A. J. (2014). Spatial-temporal analysis of Cache Valley virus (Bunyaviridae: Orthobunyavirus) infection in anopheline and culicine mosquitoes (diptera: Culicidae) in the northeastern United States, 1997-2012. *Vector-Borne and Zoonotic Diseases, 14*, 763-773. | Ngo, K. A., Maffei, J. G., Dupuis Ii, A. P., Kauffman, E. B., Backenson, P. B., & Kramer, L. D. (2006). Isolation of Bunyamwera serogroup viruses (bunyaviridae, orthobunyavirus) in New York state. *Journal of Medical Entomology, 43*, 1004-1009. | Main, A. J. (1981). Arbovirus surveillance in Connecticut. IV. Bunyamwera group. *Mosquito News*, 41, 490-494. |  |
| *Aedes japonicus* | Vector | Anderson, J.F., Armstrong, P.M., Misencik, M.J., Bransfield, A.B., Andreadis, T.G., & Molaei, G. (2018). Seasonal distribution, blood-feeding habits, and viruses of mosquitoes in an open-faced quarry in Connecticut, 2010 and 2011. Journal of the *American Mosquito Control Association*, 34, 1–10. | Yang, F., Chan, K., Marek, P.E., Armstrong, P.M., Liu, P., Bova, J.E., Bernick, J.N., McMillan, B.E., Weidlich, B.G., Paulson, S.L. (2018). Cache Valley Virus in *Aedes japonicus japonicus* mosquitoes, Appalachian Region, United States. *Emerging Infectious Diseases*, 24,553-557. | Andreadis, T. G., Armstrong, P. M., Anderson, J. F., & Main, A. J. (2014). Spatial-temporal analysis of Cache Valley virus (Bunyaviridae: Orthobunyavirus) infection in anopheline and culicine mosquitoes (diptera: Culicidae) in the northeastern United States, 1997-2012. *Vector-Borne and Zoonotic Diseases*, 14, 763-773. |  |
| *Aedes scapularis* | Vector | Belle, E. A., King, S. D., Griffiths, B. B., Grant, L. S. (1980). Epidemiological investigation for arboviruses in Jamaica, West Indies. American *Journal of Tropical Medicine and Hygiene* 29, 667-675. | Scherer, W. F., Campillo-Sainz, C., Dickerman, R. W., Diaz-Najera, A., & Madalengoitia, J. (1967). Isolation of Tlacotalpan virus, a new Bunyamwera-group virus from Mexican mosquitoes. *American Journal of Tropical Medicine and Hygiene, 16*, 79-91. |  |  |
| *Aedes serratus* | Vector | Scherer, W. F., Campillo-Sainz, C., Dickerman, R. W., Diaz-Najera, A., & Madalengoitia, J. (1967). Isolation of Tlacotalpan virus, a new Bunyamwera-group virus from Mexican mosquitoes. *American Journal of Tropical Medicine and Hygiene, 16*, 79-91. |  |  |  |
| *Aedes sollicitans* | Vector | Andreadis, T. G., Armstrong, P. M., Anderson, J. F., & Main, A. J. (2014). Spatial-temporal analysis of Cache Valley virus (Bunyaviridae: Orthobunyavirus) infection in anopheline and culicine mosquitoes (diptera: Culicidae) in the northeastern United States, 1997-2012. *Vector-Borne and Zoonotic Diseases, 14*, 763-773. | Anderson, J.F., Armstrong, P.M., Misencik, M.J., Bransfield, A.B., Andreadis, T.G., & Molaei, G. (2018). Seasonal distribution, blood-feeding habits, and viruses of mosquitoes in an open-faced quarry in Connecticut, 2010 and 2011. Journal of the *American Mosquito Control Association*, 34, 1–10. | Farfan-Ale, J., Lorono-Pino, M., Garcia-Rejon, J., Soto, V., Lin, M., Staley, M., . . . Blitvich, B. J. (2010). Detection of flaviviruses and orthobunyaviruses in mosquitoes in the Yucatan peninsula of Mexico in 2008. *Vector Borne Zoonotic Dis*, 10, 777-783. |  |
| *Aedes sticticus* | Vector | Andreadis, T. G., Armstrong, P. M., Anderson, J. F., & Main, A. J. (2014). Spatial-temporal analysis of Cache Valley virus (Bunyaviridae: Orthobunyavirus) infection in anopheline and culicine mosquitoes (diptera: Culicidae) in the northeastern United States, 1997-2012. *Vector-Borne and Zoonotic Diseases, 14*, 763-773. | Ngo, K. A., Maffei, J. G., Dupuis Ii, A. P., Kauffman, E. B., Backenson, P. B., & Kramer, L. D. (2006). Isolation of Bunyamwera serogroup viruses (bunyaviridae, orthobunyavirus) in New York state. *Journal of Medical Entomology, 43*, 1004-1009. | Main, A. J. (1981). Arbovirus surveillance in Connecticut. IV. Bunyamwera group. *Mosquito News*, 41, 490-494. |  |
| *Aedes stimulans* | Vector | Andreadis, T. G., Armstrong, P. M., Anderson, J. F., & Main, A. J. (2014). Spatial-temporal analysis of Cache Valley virus (Bunyaviridae: Orthobunyavirus) infection in anopheline and culicine mosquitoes (diptera: Culicidae) in the northeastern United States, 1997-2012. *Vector-Borne and Zoonotic Diseases, 14*, 763-773. | Ngo, K. A., Maffei, J. G., Dupuis Ii, A. P., Kauffman, E. B., Backenson, P. B., & Kramer, L. D. (2006). Isolation of Bunyamwera serogroup viruses (bunyaviridae, orthobunyavirus) in New York state. *Journal of Medical Entomology, 43*, 1004-1009. | Main, A. J. (1981). Arbovirus surveillance in Connecticut. IV. Bunyamwera group. *Mosquito News*, 41, 490-494. |  |
| *Aedes taeniorhynchus* | Vector | Andreadis, T. G., Armstrong, P. M., Anderson, J. F., & Main, A. J. (2014). Spatial-temporal analysis of Cache Valley virus (Bunyaviridae: Orthobunyavirus) infection in anopheline and culicine mosquitoes (diptera: Culicidae) in the northeastern United States, 1997-2012. *Vector-Borne and Zoonotic Diseases, 14*, 763-773. | Farfan-Ale, J., Lorono-Pino, M., Garcia-Rejon, J., Soto, V., Lin, M., Staley, M., . . . Blitvich, B. J. (2010). Detection of flaviviruses and orthobunyaviruses in mosquitoes in the Yucatan peninsula of Mexico in 2008. *Vector Borne Zoonotic Dis, 10*, 777-783. | Belle, E. A., King, S. D., Griffiths, B. B., Grant, L. S. (1980). Epidemiological investigation for arboviruses in Jamaica, West Indies. American *Journal of Tropical Medicine and Hygiene* 29, 667-675. |  |
| *Aedes triseriatus* | Vector | Andreadis, T. G., Armstrong, P. M., Anderson, J. F., & Main, A. J. (2014). Spatial-temporal analysis of Cache Valley virus (Bunyaviridae: Orthobunyavirus) infection in anopheline and culicine mosquitoes (diptera: Culicidae) in the northeastern United States, 1997-2012. *Vector-Borne and Zoonotic Diseases, 14*, 763-773. | Edwards, J. F., Higgs, S., & Beaty, B. J. (1998). Mosquito feeding-induced enhancement of Cache Valley virus (bunyaviridae) infection in mice. *Journal of Medical Entomology, 35*, 261-265. | Ngo, K. A., Maffei, J. G., Dupuis Ii, A. P., Kauffman, E. B., Backenson, P. B., & Kramer, L. D. (2006). Isolation of Bunyamwera serogroup viruses (bunyaviridae, orthobunyavirus) in New York state. *Journal of Medical Entomology*, 43, 1004-1009. |  |
| *Aedes trivittatus* | Vector | Andreadis, T. G., Armstrong, P. M., Anderson, J. F., & Main, A. J. (2014). Spatial-temporal analysis of Cache Valley virus (Bunyaviridae: Orthobunyavirus) infection in anopheline and culicine mosquitoes (diptera: Culicidae) in the northeastern United States, 1997-2012. *Vector-Borne and Zoonotic Diseases, 14*, 763-773. | Anderson, J.F., Armstrong, P.M., Misencik, M.J., Bransfield, A.B., Andreadis, T.G., & Molaei, G. (2018). Seasonal distribution, blood-feeding habits, and viruses of mosquitoes in an open-faced quarry in Connecticut, 2010 and 2011. Journal of the *American Mosquito Control Association*, 34, 1–10. | Anderson, J. F., Main, A. J., Armstrong, P. M., Andreadis, T. G., & Ferrandino, F. J. (2015). Arboviruses in North Dakota, 2003-2006. *American Journal of Tropical Medicine and Hygiene*, 92, 377-393. |  |
| *Aedes vexans* | Vector | Andreadis, T. G., Armstrong, P. M., Anderson, J. F., & Main, A. J. (2014). Spatial-temporal analysis of Cache Valley virus (Bunyaviridae: Orthobunyavirus) infection in anopheline and culicine mosquitoes (diptera: Culicidae) in the northeastern United States, 1997-2012. *Vector-Borne and Zoonotic Diseases, 14*, 763-773. | Anderson, J.F., Armstrong, P.M., Misencik, M.J., Bransfield, A.B., Andreadis, T.G., & Molaei, G. (2018). Seasonal distribution, blood-feeding habits, and viruses of mosquitoes in an open-faced quarry in Connecticut, 2010 and 2011. Journal of the *American Mosquito Control Association*, 34, 1–10. | Buescher, E. L., Byrne, R. J., Clarke, G. C., Gould, D. J., Russell, P. K., Scheider, F. G., & Yuill, T. M. (1970). Cache Valley virus in the Delmarva peninsula. I. virologic and serologic evidence of infection. American *Journal of Tropical Medicine and Hygiene*, 19, 493-502. |  |
| *Anopheles albimanus* | Vector | Farfan-Ale, J., Lorono-Pino, M., Garcia-Rejon, J., Soto, V., Lin, M., Staley, M., . . . Blitvich, B. J. (2010). Detection of flaviviruses and orthobunyaviruses in mosquitoes in the Yucatan peninsula of Mexico in 2008. *Vector Borne Zoonotic Dis, 10*, 777-783. | Belle, E. A., King, S. D., Griffiths, B. B., Grant, L. S. (1980). Epidemiological investigation for arboviruses in Jamaica, West Indies. American *Journal of Tropical Medicine and Hygiene* 29, 667-675. |  |  |
| *Anopheles crucians* | Vector | Anderson, J.F., Armstrong, P.M., Misencik, M.J., Bransfield, A.B., Andreadis, T.G., & Molaei, G. (2018). Seasonal distribution, blood-feeding habits, and viruses of mosquitoes in an open-faced quarry in Connecticut, 2010 and 2011. Journal of the *American Mosquito Control Association*, 34, 1–10. | Buescher, E. L., Byrne, R. J., Clarke, G. C., Gould, D. J., Russell, P. K., Scheider, F. G., & Yuill, T. M. (1970). Cache Valley virus in the Delmarva peninsula. I. virologic and serologic evidence of infection. *American Journal of Tropical Medicine and Hygiene, 19*, 493-502. |  |  |
| *Anopheles freeborni* | Vector | Elbel, R. E., Crane, G. T., Stipe, L. E., Nosdol, G. B. V., & Smart, K. L. (1971). Arbovirus isolations from mosquitoes collected at Callao, Utah 1966 and 1967. *Mosquito News, 31*, 61-68. | Crane, G. T., Elbel, R. E., Francy, D. B., & Calisher, C. H. (1983). Arboviruses from western Utah, USA, 1967-1976. *Journal of Medical Entomology, 20*, 194-300. |  |  |
| *Anopheles grabhami* | Vector | Belle, E. A., King, S. D., Griffiths, B. B., Grant, L. S. (1980). Epidemiological investigation for arboviruses in Jamaica, West Indies. *American Journal of Tropical Medicine and Hygiene* 29, 667-675. |  |  | This is a South American species it is excluded due to low occurrence count in North America |
| *Anopheles punctipennis* | Vector | Andreadis, T. G., Armstrong, P. M., Anderson, J. F., & Main, A. J. (2014). Spatial-temporal analysis of Cache Valley virus (Bunyaviridae: Orthobunyavirus) infection in anopheline and culicine mosquitoes (diptera: Culicidae) in the northeastern United States, 1997-2012. *Vector-Borne and Zoonotic Diseases, 14*, 763-773. | Anderson, J.F., Armstrong, P.M., Misencik, M.J., Bransfield, A.B., Andreadis, T.G., & Molaei, G. (2018). Seasonal distribution, blood-feeding habits, and viruses of mosquitoes in an open-faced quarry in Connecticut, 2010 and 2011. Journal of the *American Mosquito Control Association*, 34, 1–10. | Ngo, K. A., Maffei, J. G., Dupuis Ii, A. P., Kauffman, E. B., Backenson, P. B., & Kramer, L. D. (2006). Isolation of Bunyamwera serogroup viruses (bunyaviridae, orthobunyavirus) in New York state. *Journal of Medical Entomology*, 43, 1004-1009. |  |
| *Anopheles quadrimaculatus* | Vector | Andreadis, T. G., Armstrong, P. M., Anderson, J. F., & Main, A. J. (2014). Spatial-temporal analysis of Cache Valley virus (Bunyaviridae: Orthobunyavirus) infection in anopheline and culicine mosquitoes (diptera: Culicidae) in the northeastern United States, 1997-2012. *Vector-Borne and Zoonotic Diseases, 14*, 763-773. | Anderson, J.F., Armstrong, P.M., Misencik, M.J., Bransfield, A.B., Andreadis, T.G., & Molaei, G. (2018). Seasonal distribution, blood-feeding habits, and viruses of mosquitoes in an open-faced quarry in Connecticut, 2010 and 2011. Journal of the *American Mosquito Control Association*, 34, 1–10. | Buescher, E. L., Byrne, R. J., Clarke, G. C., Gould, D. J., Russell, P. K., Scheider, F. G., & Yuill, T. M. (1970). Cache Valley virus in the Delmarva peninsula. I. virologic and serologic evidence of infection. *American Journal of Tropical Medicine and Hygiene*, 19, 493-502. |  |
| *Anopheles walkeri* | Vector | Andreadis, T. G., Armstrong, P. M., Anderson, J. F., & Main, A. J. (2014). Spatial-temporal analysis of Cache Valley virus (Bunyaviridae: Orthobunyavirus) infection in anopheline and culicine mosquitoes (diptera: Culicidae) in the northeastern United States, 1997-2012. *Vector-Borne and Zoonotic Diseases, 14*, 763-773. | Ngo, K. A., Maffei, J. G., Dupuis Ii, A. P., Kauffman, E. B., Backenson, P. B., & Kramer, L. D. (2006). Isolation of Bunyamwera serogroup viruses (bunyaviridae, orthobunyavirus) in New York state. *Journal of Medical Entomology, 43*, 1004-1009. | Main, A. J. (1981). Arbovirus surveillance in Connecticut. IV. Bunyamwera group. *Mosquito News*, 41, 490-494. |  |
| *Cervus elephanus* | Host | Eldridge, B. F., Calisher, C. H., Fryer, J. L., Bright, L., & Hobbs, D. J. (1987). Serological evidence of California serogroup virus activity in Oregon. *Journal of Wildlife Diseases, 23*, 199-204. |  |  |  |
| *Coquillettidia perturbans* | Vector | Andreadis, T. G., Armstrong, P. M., Anderson, J. F., & Main, A. J. (2014). Spatial-temporal analysis of Cache Valley virus (Bunyaviridae: Orthobunyavirus) infection in anopheline and culicine mosquitoes (diptera: Culicidae) in the northeastern United States, 1997-2012. *Vector-Borne and Zoonotic Diseases, 14*, 763-773. | Anderson, J.F., Armstrong, P.M., Misencik, M.J., Bransfield, A.B., Andreadis, T.G., & Molaei, G. (2018). Seasonal distribution, blood-feeding habits, and viruses of mosquitoes in an open-faced quarry in Connecticut, 2010 and 2011. *Journal of the American Mosquito Control Association*, 34, 1–10. | Ngo, K. A., Maffei, J. G., Dupuis Ii, A. P., Kauffman, E. B., Backenson, P. B., & Kramer, L. D. (2006). Isolation of Bunyamwera serogroup viruses (bunyaviridae, orthobunyavirus) in New York state. *Journal of Medical Entomology*, 43, 1004-1009. |  |
| *Coquillettidia venezuelensis* | Vector | Farfan-Ale, J., Lorono-Pino, M., Garcia-Rejon, J., Soto, V., Lin, M., Staley, M., . . . Blitvich, B. J. (2010). Detection of flaviviruses and orthobunyaviruses in mosquitoes in the Yucatan peninsula of Mexico in 2008. *Vector Borne Zoonotic Dis, 10*, 777-783. |  |  | This is a South American species it is excluded due to low occurrence count in North America |
| *Culex corniger* | Vector | Belle, E. A., King, S. D., Griffiths, B. B., Grant, L. S. (1980). Epidemiological investigation for arboviruses in Jamaica, West Indies. *American Journal of Tropical Medicine and Hygiene* 29, 667-675. | Scherer, W. F., Campillo-Sainz, C., Dickerman, R. W., Diaz-Najera, A., & Madalengoitia, J. (1967). Isolation of Tlacotalpan virus, a new Bunyamwera-group virus from Mexican mosquitoes. *American Journal of Tropical Medicine and Hygiene, 16*, 79-91. |  |  |
| *Culex fatigans* | Vector | Belle, E. A., King, S. D., Griffiths, B. B., Grant, L. S. (1980). Epidemiological investigation for arboviruses in Jamaica, West Indies. *American Journal of Tropical Medicine and Hygiene* 29, 667-675. |  |  | Excluded due to it being reclassified into *Culex quinquefasciatus* |
| *Culex nigripalpus* | Vector | Belle, E. A., King, S. D., Griffiths, B. B., Grant, L. S. (1980). Epidemiological investigation for arboviruses in Jamaica, West Indies. *American Journal of Tropical Medicine and Hygiene* 29, 667-675. | Farfan-Ale, J., Lorono-Pino, M., Garcia-Rejon, J., Soto, V., Lin, M., Staley, M., . . . Blitvich, B. J. (2010). Detection of flaviviruses and orthobunyaviruses in mosquitoes in the Yucatan peninsula of Mexico in 2008. *Vector Borne Zoonotic Dis, 10*, 777-783. | Ortiz, D. I., Wozniak, A., Tolson, M. W., & Turner, P. E. (2005). Arbovirus circulation, temporal distribution, and abundance of mosquito species in two Carolina bay habitats. *Vector-Borne and Zoonotic Diseases*, 5, 20-32. |  |
| *Culex pilosus* | Vector | Belle, E. A., King, S. D., Griffiths, B. B., Grant, L. S. (1980). Epidemiological investigation for arboviruses in Jamaica, West Indies. *American Journal of Tropical Medicine and Hygiene* 29, 667-675. |  |  |  |
| *Culex pipiens* | Vector | Andreadis, T. G., Armstrong, P. M., Anderson, J. F., & Main, A. J. (2014). Spatial-temporal analysis of Cache Valley virus (Bunyaviridae: Orthobunyavirus) infection in anopheline and culicine mosquitoes (diptera: Culicidae) in the northeastern United States, 1997-2012. *Vector-Borne and Zoonotic Diseases, 14*, 763-773. | Anderson, J.F., Armstrong, P.M., Misencik, M.J., Bransfield, A.B., Andreadis, T.G., & Molaei, G. (2018). Seasonal distribution, blood-feeding habits, and viruses of mosquitoes in an open-faced quarry in Connecticut, 2010 and 2011. *Journal of the American Mosquito Control Association*, 34, 1–10. | Edwards, J. F., Higgs, S., & Beaty, B. J. (1998). Mosquito feeding-induced enhancement of Cache Valley virus (bunyaviridae) infection in mice. *Journal of Medical Entomology*, 35, 261-265. |  |
| *Culex quinquefasciatus* | Vector | Farfan-Ale, J., Lorono-Pino, M., Garcia-Rejon, J., Soto, V., Lin, M., Staley, M., . . . Blitvich, B. J. (2010). Detection of flaviviruses and orthobunyaviruses in mosquitoes in the Yucatan peninsula of Mexico in 2008. *Vector Borne Zoonotic Dis, 10*, 777-783. | Scherer, W. F., Campillo-Sainz, C., Dickerman, R. W., Diaz-Najera, A., & Madalengoitia, J. (1967). Isolation of Tlacotalpan virus, a new Bunyamwera-group virus from Mexican mosquitoes. *American Journal of Tropical Medicine and Hygiene, 16*, 79-91. | Kokernot, R. H., Hayes, J., Boyd, K. R., & Sullivan, P. S. (1974). Arbovirus studies in Houston, Texas, 1968-1970. *Journal of Medical Entomology*, 11, 419-425. |  |
| *Culex restuans* | Vector | Andreadis, T. G., Armstrong, P. M., Anderson, J. F., & Main, A. J. (2014). Spatial-temporal analysis of Cache Valley virus (Bunyaviridae: Orthobunyavirus) infection in anopheline and culicine mosquitoes (diptera: Culicidae) in the northeastern United States, 1997-2012. *Vector-Borne and Zoonotic Diseases, 14*, 763-773. | Anderson, J.F., Armstrong, P.M., Misencik, M.J., Bransfield, A.B., Andreadis, T.G., & Molaei, G. (2018). Seasonal distribution, blood-feeding habits, and viruses of mosquitoes in an open-faced quarry in Connecticut, 2010 and 2011. *Journal of the American Mosquito Control Association*, 34, 1–10. | Main, A. J. (1981). Arbovirus surveillance in Connecticut. IV. Bunyamwera group. *Mosquito News*, 41, 490-494. |  |
| *Culex salinarius* | Vector | Andreadis, T. G., Armstrong, P. M., Anderson, J. F., & Main, A. J. (2014). Spatial-temporal analysis of Cache Valley virus (Bunyaviridae: Orthobunyavirus) infection in anopheline and culicine mosquitoes (diptera: Culicidae) in the northeastern United States, 1997-2012. *Vector-Borne and Zoonotic Diseases, 14*, 763-773. | Anderson, J.F., Armstrong, P.M., Misencik, M.J., Bransfield, A.B., Andreadis, T.G., & Molaei, G. (2018). Seasonal distribution, blood-feeding habits, and viruses of mosquitoes in an open-faced quarry in Connecticut, 2010 and 2011. *Journal of the American Mosquito Control Association*, 34, 1–10. | Buescher, E. L., Byrne, R. J., Clarke, G. C., Gould, D. J., Russell, P. K., Scheider, F. G., & Yuill, T. M. (1970). Cache Valley virus in the Delmarva peninsula. I. virologic and serologic evidence of infection. *American Journal of Tropical Medicine and Hygiene*, 19, 493-502. |  |
| *Culex tarsalis* | Vector | Anderson, J. F., Main, A. J., Armstrong, P. M., Andreadis, T. G., & Ferrandino, F. J. (2015). Arboviruses in North Dakota, 2003-2006. *American Journal of Tropical Medicine and Hygiene, 92*, 377-393. | Pabbaraju, K., Ho, K. C. F., Wong, S., Fox, J. D., Kaplen, B., Tyler, S., . . . Tilley, P. A. G. (2009). Surveillance of mosquito-borne viruses in Alberta using reverse transcription polymerase chain reaction with generic primers. *Journal of Medical Entomology, 46*, 640-648. | Farfan-Ale, J., Lorono-Pino, M. A., Garcia-Rejon, J., Hovav, E., Powers, A. M., Lin, M., . . . Blitvich, B. J. (2009). Detection of RNA from a novel West Nile-like virus and high prevalence of an insect-specific flavivirus in mosquitoes in the Yucatan peninsula of Mexico. *American Journal of Tropical Medicine and Hygiene*, 80, 85-95. |  |
| *Culiseta inornata* | Vector | Ngo, K. A., Maffei, J. G., Dupuis Ii, A. P., Kauffman, E. B., Backenson, P. B., & Kramer, L. D. (2006). Isolation of Bunyamwera serogroup viruses (bunyaviridae, orthobunyavirus) in New York state. *Journal of Medical Entomology, 43*, 1004-1009. | Main, A. J. (1981). Arbovirus surveillance in Connecticut. IV. Bunyamwera group. *Mosquito News, 41*, 490-494. | Anderson, J. F., Main, A. J., Armstrong, P. M., Andreadis, T. G., & Ferrandino, F. J. (2015). Arboviruses in North Dakota, 2003-2006. *American Journal of Tropical Medicine and Hygiene*, 92, 377-393. |  |
| *Culiseta melanura* | Vector | Andreadis, T. G., Armstrong, P. M., Anderson, J. F., & Main, A. J. (2014). Spatial-temporal analysis of Cache Valley virus (Bunyaviridae: Orthobunyavirus) infection in anopheline and culicine mosquitoes (diptera: Culicidae) in the northeastern United States, 1997-2012. *Vector-Borne and Zoonotic Diseases, 14*, 763-773. | Anderson, J.F., Armstrong, P.M., Misencik, M.J., Bransfield, A.B., Andreadis, T.G., & Molaei, G. (2018). Seasonal distribution, blood-feeding habits, and viruses of mosquitoes in an open-faced quarry in Connecticut, 2010 and 2011. *Journal of the American Mosquito Control Association*, 34, 1–10. | Buescher, E. L., Byrne, R. J., Clarke, G. C., Gould, D. J., Russell, P. K., Scheider, F. G., & Yuill, T. M. (1970). Cache Valley virus in the Delmarva peninsula. I. virologic and serologic evidence of infection. *American Journal of Tropical Medicine and Hygiene*, 19, 493-502. |  |
| *Lepus california* | Host | Aguirre, A. A., McLean, R. G., Cook, R. S., & Quan, T. J. (1992). Serologic survey for selected arboviruses and other potential pathogens in wildlife from Mexico. *Journal of Wildlife Diseases, 28*, 435-442. |  |  |  |
| *Mansonia perturbans* | Vector | Anderson, J.F., Armstrong, P.M., Misencik, M.J., Bransfield, A.B., Andreadis, T.G., & Molaei, G. (2018). Seasonal distribution, blood-feeding habits, and viruses of mosquitoes in an open-faced quarry in Connecticut, 2010 and 2011. *Journal of the American Mosquito Control Association*, 34, 1–10. |  |  | Excluded- Now considered to be part of the Coquillettidia perturbans group |
| *Mansonia titillans* | Vector | Farfan-Ale, J., Lorono-Pino, M., Garcia-Rejon, J., Soto, V., Lin, M., Staley, M., . . . Blitvich, B. J. (2010). Detection of flaviviruses and orthobunyaviruses in mosquitoes in the Yucatan peninsula of Mexico in 2008. *Vector Borne Zoonotic Dis, 10*, 777-783. | Belle, E. A., King, S. D., Griffiths, B. B., Grant, L. S. (1980). Epidemiological investigation for arboviruses in Jamaica, West Indies. *American Journal of Tropical Medicine and Hygiene* 29, 667-675. |  |  |
| *Odocoileus hemionus* | Host | McLean, R. G., Kirk, L. J., Shriner, R. B., Cook, P. D., Myers, E. E., Gill, J. S., & Campos, E. G. (1996). The role of deer as a possible reservoir host of potosi virus, a newly recognized arbovirus in the United States. *Journal of Wildlife Diseases, 32*, 444-452. | Eldridge, B. F., Calisher, C. H., Fryer, J. L., Bright, L., & Hobbs, D. J. (1987). Serological evidence of California serogroup virus activity in Oregon. *Journal of Wildlife Diseases, 23*, 199-204. |  |  |
| *Odocoileus virginiana* | Host | Aguirre, A. A., McLean, R. G., Cook, R. S., & Quan, T. J. (1992). Serologic survey for selected arboviruses and other potential pathogens in wildlife from Mexico. *Journal of Wildlife Diseases, 28*, 435-442. | Blackmore, C. G. M., & Grimstad, P. R. (1998). Cache Valley and Potosi viruses (Bunyaviridae) in white-tailed deer (Odocoileus virginianus): Experimental infections and antibody prevalence in natural populations. *American Journal of Tropical Medicine and Hygiene, 59*, 704-709. 196 | McLean, R. G., Kirk, L. J., Shriner, R. B., Cook, P. D., Myers, E. E., Gill, J. S., & Campos, E. G. (1996). The role of deer as a possible reservoir host of potosi virus, a newly recognized arbovirus in the United States. *Journal of Wildlife Diseases*, 32, 444-452. |  |
| *Ovis canadensis* | Host |  |  |  | Nobody has screened wild Ovis Species for CVV, but every Ovis species that has been screened has shown to be competent. |
| *Ovis dalli* | Host |  |  |  | Nobody has screened wild Ovis Species for CVV, but every Ovis species that has been screened has shown to be competent. |
| *Procyon lotor* | Host | Kokernot, R. H., Hayes, J., Tempelis, C. H., Chan, D. H., Boyd, K. R., Anderson, R. J. (1969b). Arbovirus studies in the Ohio-Mississippi Basin, 1964-1967. IV. Cache Valley virus. *The American Journal of tropical medicine and hygiene*. 18, 768-773 | Buescher, E. L., Byrne, R. J., Clarke, G. C., Gould, D. J., Russell, P. K., Scheider, F. G., & Yuill, T. M. (1970). Cache Valley virus in the Delmarva peninsula. I. virologic and serologic evidence of infection. *American Journal of Tropical Medicine and Hygiene, 19*, 493-502. |  |  |
| *Psorophora cingulata* | Vector | Galindo, P., Srihongse, S., De Rodaniche, E., & Grayson, M. A. (1966). An ecological survey for arboviruses in Almirante, Panama, 1959-1962. *American Journal of Tropical Medicine and Hygiene*, 15, 385-400. |  |  | This is a South American species it is excluded due to low occurrence count in North America |
| *Psorophora ferox* | Vector | Andreadis, T. G., Armstrong, P. M., Anderson, J. F., & Main, A. J. (2014). Spatial-temporal analysis of Cache Valley virus (Bunyaviridae: Orthobunyavirus) infection in anopheline and culicine mosquitoes (diptera: Culicidae) in the northeastern United States, 1997-2012. *Vector-Borne and Zoonotic Diseases, 14*, 763-773. | Farfan-Ale, J., Lorono-Pino, M., Garcia-Rejon, J., Soto, V., Lin, M., Staley, M., . . . Blitvich, B. J. (2010). Detection of flaviviruses and orthobunyaviruses in mosquitoes in the Yucatan peninsula of Mexico in 2008. *Vector Borne Zoonotic Dis, 10*, 777-783. | Ngo, K. A., Maffei, J. G., Dupuis Ii, A. P., Kauffman, E. B., Backenson, P. B., & Kramer, L. D. (2006). Isolation of Bunyamwera serogroup viruses (bunyaviridae, orthobunyavirus) in New York state. *Journal of Medical Entomology*, 43, 1004-1009. |  |
| *Sylvilagus floridianus* | Host | Blackmore, C. G., & Grimstad, P. R. (2008). Evaluation of the eastern cottontail Sylvilagus floridanus as an amplifying vertebrate host for Cache Valley virus (Bunyaviridae) in Indiana. *Journal of Wildlife Diseases*, 44, 188-192. |  |  |  |
| *Vulpes macrotis* | Host | Miller, D. S., Covell, D. F., McLean, R. G., Adrian, W. J., Niezgoda, M., Gustafson, J. M., . . . Quan, T. J. (2000). Serologic survey for selected infectious disease agents in swift and kit foxes from the western United States. *Journal of Wildlife Diseases*, 36, 798-805. |  |  |  |
| *Vulpes velox* | Host | Miller, D. S., Covell, D. F., McLean, R. G., Adrian, W. J., Niezgoda, M., Gustafson, J. M., . . . Quan, T. J. (2000). Serologic survey for selected infectious disease agents in swift and kit foxes from the western United States. *Journal of Wildlife Diseases*, 36, 798-805. |  |  |  |
| *Marmota monax* | Host | Kokernot, R. H., Hayes, J., Tempelis, C. H., Chan, D. H., Boyd, K. R., Anderson, R. J. (1969b). Arbovirus studies in the Ohio-Mississippi Basin, 1964-1967. IV. Cache Valley virus. *The American Journal of tropical medicine and hygiene*. 18, 768-773 | Buescher, E. L., Byrne, R. J., Clarke, G. C., Gould, D. J., Russell, P. K., Scheider, F. G., & Yuill, T. M. (1970). Cache Valley virus in the Delmarva peninsula. I. virologic and serologic evidence of infection. *American Journal of Tropical Medicine and Hygiene, 19*, 493-502. |  |  |
| *Urocyon cinereoargenteus* | Host | Buescher, E. L., Byrne, R. J., Clarke, G. C., Gould, D. J., Russell, P. K., Scheider, F. G., & Yuill, T. M. (1970). Cache Valley virus in the Delmarva peninsula. I. virologic and serologic evidence of infection. *American Journal of Tropical Medicine and Hygiene, 19*, 493-502. |  |  | Excluded due multiple studies not finding any seropositive animals, and only 1 study having any sero-positives and that study having only 1 individual positive |

Table S2

Cache Valley virus location data. Locations were derived from literature review and online databases. Online databases included GenBank, World Reference Center for Emerging Viruses and Arboviruses (WRCEVA), and the Center for disease Control and Prevention (CDC) Arboviral Reference Collection. The literature review was conducted using the search term “Cache Valley virus” using the online databases of Google Scholar and PubMed as well as using all papers listed in the large review paper from Waddell et al. 2019. The literature search was conducted until December 2022, and the query period encompassed 1959-2022. Papers from outside north America were excluded due to the possibility that they were misdiagnosed as maguari or other related viruses. Latitude and Longitude data were georeferenced from the descriptions in the paper. Inclusion of location was dependent upon how specific the location described in the paper was. Papers with less specific location data (i.e., only the state was given (e.g., Texas), or generic location data (e.g., Western Idaho)) were not included. Papers that had specific locations (i.e., ranch across from the V. Carranza Dam) were included.

| Paper/ Isolate ID | Species | Location | Latitude | Longitude |
| --- | --- | --- | --- | --- |
| Centers for Disease Control and Prevention, Arbovirus Reference Collection (Isolate ID: 37-1784) | *Culiseta inornata* | Callao, Juab County, UT | 39.89772 | -113.709 |
| Centers for Disease Control and Prevention, Arbovirus Reference Collection (Isolate ID: 84-4046) | *Ae. taeniorhynchus/ sollicitans* | Fort Detrick, Frederick, MD | 39.43429 | -77.4278 |
| Aguirre, A. A., McLean, R. G., Cook, R. S., & Quan, T. J. (1992). Serologic survey for selected arboviruses and other potential pathogens in wildlife from Mexico. Journal of Wildlife Diseases, 28, 435-442. | Deer, Jackrabbits | V. Carranza dam, Coahuila México | 27.51972 | -100.606 |
| Andre, R. G., Rowley, W. A., Wong, Y. W., & Dorsey, D. C. (1985). Surveillance of arbovirus activity in Iowa, USA, 1978-1980. Journal of Medical Entomology, 22, 58-63. | Various Mosquitos | Council Bluffs, Iowa | 41.20032 | -95.8846 |
| Andre, R. G., Rowley, W. A., Wong, Y. W., & Dorsey, D. C. (1985). Surveillance of arbovirus activity in Iowa, USA, 1978-1980. Journal of Medical Entomology, 22, 58-63. | Various Mosquitos | Davenport, Iowa | 41.52364 | -90.5776 |
| Anslow, R. O., Thompson, W. H., Thompson, P. H., Defoliart, G. R., Papadopoulos, O., & Hanson, R. P. (1969). Isolation of Bunyamwera-group viruses from Wisconsin mosquitoes. American Journal of Tropical Medicine and Hygiene, 18, 599-608. | *Ae. communis* | Black River falls | 44.29468 | -90.8515 |
| Anslow, R. O., Thompson, W. H., Thompson, P. H., Defoliart, G. R., Papadopoulos, O., & Hanson, R. P. (1969). Isolation of Bunyamwera-group viruses from Wisconsin mosquitoes. American Journal of Tropical Medicine and Hygiene, 18, 599-608. | *Ae. vexans* | University Arboretum, Madison | 43.04132 | -89.4305 |
| Anslow, R. O., Thompson, W. H., Thompson, P. H., Defoliart, G. R., Papadopoulos, O., & Hanson, R. P. (1969). Isolation of Bunyamwera-group viruses from Wisconsin mosquitoes. American Journal of Tropical Medicine and Hygiene, 18, 599-608. | *Ae. vexans* | Mazomanie Wildlife Area | 43.24097 | -89.7392 |
| Anslow, R. O., Thompson, W. H., Thompson, P. H., Defoliart, G. R., Papadopoulos, O., & Hanson, R. P. (1969). Isolation of Bunyamwera-group viruses from Wisconsin mosquitoes. American Journal of Tropical Medicine and Hygiene, 18, 599-608. | *Mansonia perturbans* | Tomahawk, Wisconsin | 45.47108 | -89.7299 |
| Centers for Disease Control and Prevention, Arbovirus Reference Collection (Isolate ID: Ar531) | *An. quadrimaculatus* | Lake Mermet Conservation Area, Massac Co. | 37.26227 | -88.8504 |
| [Armstrong, P.M., Anderson, J.F., Farajollahi, A., Healy, S.P., Unlu, I., Crepeau, T.N., Gaugler, R., Fonseca, D.M., & Andreadis, T.G. (2013). Isolations of Cache Valley virus from Aedes albopictus (Diptera: Culicidae) in New Jersey and evaluation of its role as a regional arbovirus vector. Journal of Medical Entomology, 50, 1310-1314](http://dx.doi.org/10.1603/ME13099) | *Ae. albopictus* | Mercer and Monmouth Counties, NJ | 40.21647 | -74.7309 |
| [Armstrong, P.M., Anderson, J.F., Farajollahi, A., Healy, S.P., Unlu, I., Crepeau, T.N., Gaugler, R., Fonseca, D.M., & Andreadis, T.G. (2013). Isolations of Cache Valley virus from Aedes albopictus (Diptera: Culicidae) in New Jersey and evaluation of its role as a regional arbovirus vector. Journal of Medical Entomology, 50, 1310-1314](http://dx.doi.org/10.1603/ME13099) | *Ae. albopictus* | Mercer and Monmouth Counties, NJ | 40.4383 | -74.1554 |
| Brockus, C. L., & Grimstad, P. R. (1999). Sequence analysis of the medium (M) segment of Cache Valley virus, with comparison to other bunyaviridae. Virus Genes, 19, 73-83. | *Culiseta inornata* | Cache Valley, Utah | 38.72498 | -109.521 |
| Brockus, C. L., & Grimstad, P. R. (2001). Comparative analysis of G1 glycoprotein-coding sequences of Cache Valley virus (Bunyaviridae: Bunyavirus) isolates. Virus Genes, 22, 133-139. | Various Mosquitos | Dennisville, NJ | 39.19229 | -74.8222 |
| Buescher, E. L., Byrne, R. J., Clarke, G. C., Gould, D. J., Russell, P. K., Scheider, F. G., & Yuill, T. M. (1970). Cache Valley virus in the Delmarva peninsula. I. virologic and serologic evidence of infection. American Journal of Tropical Medicine and Hygiene, 19, 493-502. | Various Mosquitoes and Wildlife | Various counties in Maryland and Virginia, as well as Assateague NWR, Assateague Island, Chincoteague Island | 37.90445 | -75.3477 |
| Burton, A. N., McLintock, J., & Francy, D. B. (1973). Isolation of St. Louis encephalitis and Cache Valley viruses from Saskatchewan mosquitoes. Canadian Journal of Public Health, 64, 368-373. | *Culiseta inornata* | Weyburn, Saskatchewon | 49.66328 | -103.853 |
| Chung, S. I., Livingston Jr, C. W., Jones, C. W., & Collisson, E. W. (1991). Cache Valley virus infection in Texas sheep flocks. Journal of the American Veterinary Medical Association, 199, 337-340. | Domestic Sheep | Texas A&M University Agricultural Research and Extension Center, San Angelo, TX | 31.54987 | -100.509 |
| Chung, S., Livingston Jr, C. W., Edwards, J. F., Crandell, R. W., Shope, R. E., Shelton, M. J., & Collisson, E. W. (1990b). Evidence that Cache Valley virus induces congenital malformations in sheep. Veterinary Microbiology, 21, 297-307. | Domestic Sheep | Texas A&M University Agricultural Research and Extension Center, San Angelo, TX | 31.54987 | -100.509 |
| Clark, G. G., Crabbs, C. L., Bailey, C. L., Calisher, C. H., & Craig Jr, G. B. (1986). Identification of Aedes campestris from New Mexico: With notes on the isolation of western equine encephalitis and other arboviruses. Journal of the American Mosquito Control Association, 2, 529-534. | *Aedes dorsalis* | White Sands Missile Range, Malpais spring | 33.28813 | -106.309 |
| Crane, G. T., Elbel, R. E., Francy, D. B., & Calisher, C. H. (1983). Arboviruses from western Utah, USA, 1967-1976. *Journal of Medical Entomology, 20*, 194-300. | *Aedes dorsalis* | Callao, Utah | 39.89772 | -113.709 |
| Crane, G. T., Elbel, R. E., Francy, D. B., & Calisher, C. H. (1983). Arboviruses from western Utah, USA, 1967-1976. *Journal of Medical Entomology, 20*, 194-300. | *Culiseta inornata* | Hickman Canyon, Utah | 40.43268 | -112.526 |
| McConnell, S., Livingston, C., J., Calisher, C., & Crandell, R. (1985). Isolation of Cache Valley virus from livestock in Texas, 1981. In: Veterinary viral diseases: their significance in south-east Asia and the western Pacific. Sydney, Australia: Academic Press. | Domestic Cows and Sheep | DeKalb, Bowie County Texas | 33.50873 | -94.6163 |
| Miller, D. S., Covell, D. F., McLean, R. G., Adrian, W. J., Niezgoda, M., Gustafson, J. M., . . . Quan, T. J. (2000). Serologic survey for selected infectious disease agents in swift and kit foxes from the western United States. Journal of Wildlife Diseases, 36, 798-805. | Foxes | Western United States | 35.25 | -119.867 |
| Miller, D. S., Covell, D. F., McLean, R. G., Adrian, W. J., Niezgoda, M., Gustafson, J. M., . . . Quan, T. J. (2000). Serologic survey for selected infectious disease agents in swift and kit foxes from the western United States. Journal of Wildlife Diseases, 36, 798-805. | Foxes | Western United States | 35.3 | -119.617 |
| Miller, D. S., Covell, D. F., McLean, R. G., Adrian, W. J., Niezgoda, M., Gustafson, J. M., . . . Quan, T. J. (2000). Serologic survey for selected infectious disease agents in swift and kit foxes from the western United States. Journal of Wildlife Diseases, 36, 798-805. | Foxes | Western United States | 35.28 | -119.467 |
| Miller, D. S., Covell, D. F., McLean, R. G., Adrian, W. J., Niezgoda, M., Gustafson, J. M., . . . Quan, T. J. (2000). Serologic survey for selected infectious disease agents in swift and kit foxes from the western United States. Journal of Wildlife Diseases, 36, 798-805. | Foxes | Western United States | 33.5 | -104.333 |
| Miller, D. S., Covell, D. F., McLean, R. G., Adrian, W. J., Niezgoda, M., Gustafson, J. M., . . . Quan, T. J. (2000). Serologic survey for selected infectious disease agents in swift and kit foxes from the western United States. Journal of Wildlife Diseases, 36, 798-805. | Foxes | Western United States | 33.0833 | -112.133 |
| Centers for Disease Control and Prevention, Arbovirus Reference Collection (Isolate ID: MPB1-1551) | *Ps. confinnis* | Palo Blanco, Tamaulipas Mexico | 26.00758 | -98.1676 |
| Centers for Disease Control and Prevention, Arbovirus Reference Collection (Isolate ID: OH AR 66-1850) | *An. punctipennis* | Mahoning County, Austintown, OH | 41.0998 | -80.7638 |
| Centers for Disease Control and Prevention, Arbovirus Reference Collection (Isolate ID: OH AR 75-1615) | *An. punctipennis* | Mentor Township, Lake County Ohio | 41.66852 | -81.3398 |
| Uehlinger, F.D., Wilkins W., Godson D.L., Drebot M.A. (2018). Seroprevalence of Cache Valley virus and related viruses in sheep and other livestock from Saskatchewan, Canada. Canadian Veterinary Journal, 4, 413-418. | Domestic Sheep | Saskatchewan, Canada | 49.97533 | -109.562 |
| Uehlinger, F.D., Wilkins W., Godson D.L., Drebot M.A. (2018). Seroprevalence of Cache Valley virus and related viruses in sheep and other livestock from Saskatchewan, Canada. Canadian Veterinary Journal, 4, 413-418. | Domestic Sheep | Saskatchewan, Canada | 53.54186 | -109.509 |
| Uehlinger, F.D., Wilkins W., Godson D.L., Drebot M.A. (2018). Seroprevalence of Cache Valley virus and related viruses in sheep and other livestock from Saskatchewan, Canada. Canadian Veterinary Journal, 4, 413-418. | Domestic Sheep | Saskatchewan, Canada | 49.20218 | -101.799 |
| Uehlinger, F.D., Wilkins W., Godson D.L., Drebot M.A. (2018). Seroprevalence of Cache Valley virus and related viruses in sheep and other livestock from Saskatchewan, Canada. Canadian Veterinary Journal, 4, 413-418. | Domestic Sheep | Saskatchewan, Canada | 52.0344 | -101.86 |
| Uehlinger, F.D., Wilkins W., Godson D.L., Drebot M.A. (2018). Seroprevalence of Cache Valley virus and related viruses in sheep and other livestock from Saskatchewan, Canada. Canadian Veterinary Journal, 4, 413-418. | Domestic Sheep | Saskatchewan, Canada | 51.76951 | -105.011 |
| Yang, F., Chan, K., Marek, P.E., Armstrong, P.M., Liu, P., Bova, J.E., Bernick, J.N., McMillan, B.E., Weidlich, B.G., Paulson, S.L. (2018a). Cache Valley Virus in Aedes japonicus japonicus mosquitoes, Appalachian Region, United States. Emerging Infectious Diseases, 24,553-557. | *Ae. japonicus* | Virginia Tech Campus | 37.2154 | -80.4238 |
| Blackmore, C. G. M., Blackmore, M. S., & Grimstad, P. R. (1998). Role of Anopheles quadrimaculatus and (diptera: Culicidae) in the transmission cycle of Cache Valley virus (Bunyaviridae: Bunyavirus) in the midwest, U.S.A. Journal of Medical Entomology, 35, 660-664. 197 | White-tailed deer | Porter Ranch, Houghton Lake Wildlife Research Area | 44.33248 | -84.7933 |
| Neitzel, D. F. & Grimstad, P. R. (1991). Serological evidence of California group and Cache Valley virus infection in Minnesota white-tailed deer. J Wildl Dis, 27(2), 230-237. | White-tailed deer | Elm Creek Park Preserve | 45.14 | -93.4368 |
| Neitzel, D. F. & Grimstad, P. R. (1991). Serological evidence of California group and Cache Valley virus infection in Minnesota white-tailed deer. J Wildl Dis, 27(2), 230-237. | White-tailed deer | Minnesota Valley NWR | 44.84586 | -93.2154 |
| Neitzel, D. F. & Grimstad, P. R. (1991). Serological evidence of California group and Cache Valley virus infection in Minnesota white-tailed deer. J Wildl Dis, 27(2), 230-237. | White-tailed deer | Carlos Avery WMA | 45.2885 | -93.1293 |
| Takeda, T., Whitehouse, C. A., Brewer, M., Gettman, A. D., & Mather, T. N. (2003). Arbovirus surveillance in Rhode Island: Assessing potential ecologic and climatic correlates. Journal of the American Mosquito Control Association, 19, 179-189. | Various Mosquitos | Rhode island | 41.41821 | -71.7937 |
| Takeda, T., Whitehouse, C. A., Brewer, M., Gettman, A. D., & Mather, T. N. (2003). Arbovirus surveillance in Rhode Island: Assessing potential ecologic and climatic correlates. Journal of the American Mosquito Control Association, 19, 179-189. | Various Mosquitos | Rhode island | 41.50077 | -71.5998 |
| Kosoy, O., Rabe, I., Geissler, A., Adjemian, J., Panella, A., Laven, J., Basile, A. J., Velez, J., Griffith, K., Wong, D., Fischer, M., Lanciotti, R. S. (2016). Serological survey for antibodies to mosquito-borne bunyaviruses among US National Park Service and US Forest Service Employees. *Vector-borne and zoonotic diseases.* 6, 191-198. | Humans | Great smokey mountain Nation al Park | 35.68661 | -83.5361 |
| Kosoy, O., Rabe, I., Geissler, A., Adjemian, J., Panella, A., Laven, J., Basile, A. J., Velez, J., Griffith, K., Wong, D., Fischer, M., Lanciotti, R. S. (2016). Serological survey for antibodies to mosquito-borne bunyaviruses among US National Park Service and US Forest Service Employees. *Vector-borne and zoonotic diseases.* 6, 191-198. | Humans | Rocky Mountain National Park | 40.37307 | -105.614 |
| Kosoy, O., Rabe, I., Geissler, A., Adjemian, J., Panella, A., Laven, J., Basile, A. J., Velez, J., Griffith, K., Wong, D., Fischer, M., Lanciotti, R. S. (2016). Serological survey for antibodies to mosquito-borne bunyaviruses among US National Park Service and US Forest Service Employees. *Vector-borne and zoonotic diseases.* 6, 191-198. | Humans | Grand Teton National Park | 43.74685 | -110.804 |
| Andreadis, T. G., Armstrong, P. M., Anderson, J. F., & Main, A. J. (2014). Spatial-temporal analysis of Cache Valley virus (Bunyaviridae: Orthobunyavirus) infection in anopheline and culicine mosquitoes (diptera: Culicidae) in the northeastern United States, 1997-2012. *Vector-Borne and Zoonotic Diseases, 14*, 763-773. | Various Mosquitos | Connecticut | 41.19376 | -73.4999 |
| Andreadis, T. G., Armstrong, P. M., Anderson, J. F., & Main, A. J. (2014). Spatial-temporal analysis of Cache Valley virus (Bunyaviridae: Orthobunyavirus) infection in anopheline and culicine mosquitoes (diptera: Culicidae) in the northeastern United States, 1997-2012. *Vector-Borne and Zoonotic Diseases, 14*, 763-773. | Various Mosquitos | Connecticut | 41.99669 | -73.3497 |
| Andreadis, T. G., Armstrong, P. M., Anderson, J. F., & Main, A. J. (2014). Spatial-temporal analysis of Cache Valley virus (Bunyaviridae: Orthobunyavirus) infection in anopheline and culicine mosquitoes (diptera: Culicidae) in the northeastern United States, 1997-2012. *Vector-Borne and Zoonotic Diseases, 14*, 763-773. | Various Mosquitos | Connecticut | 41.34292 | -71.8644 |
| Andreadis, T. G., Armstrong, P. M., Anderson, J. F., & Main, A. J. (2014). Spatial-temporal analysis of Cache Valley virus (Bunyaviridae: Orthobunyavirus) infection in anopheline and culicine mosquitoes (diptera: Culicidae) in the northeastern United States, 1997-2012. *Vector-Borne and Zoonotic Diseases, 14*, 763-773. | Various Mosquitos | Connecticut | 41.64282 | -71.9403 |
| Andreadis, T. G., Armstrong, P. M., Anderson, J. F., & Main, A. J. (2014). Spatial-temporal analysis of Cache Valley virus (Bunyaviridae: Orthobunyavirus) infection in anopheline and culicine mosquitoes (diptera: Culicidae) in the northeastern United States, 1997-2012. *Vector-Borne and Zoonotic Diseases, 14*, 763-773. | Various Mosquitos | Connecticut | 41.74456 | -72.6898 |
| Jose A. Farfan-Ale, Maria A. Loroño-Pino, Julian E. Garcia-Rejon, Victor Soto, Ming Lin, Molly Staley, Karin S. Dorman, Lyric C. Bartholomay, Einat Hovav, and Bradley J. Blitvich. Detection of Flaviviruses and Orthobunyaviruses in Mosquitoes in the Yucatan Peninsula of Mexico in 2008. Vector-Borne and Zoonotic Diseases 2010 10:8, 777-783 | Various Mosquitos | Yucatan | 20.85764 | -90.3733 |

Table S3

Descriptions of the data layers from Chelsa Climate. All layers were tested for correlation via Pearson’s Correlation Coefficient and those that were highly correlated were removed. Also stated is whether each variable was included in the model selection.

| Name | Description | Units | Inclusion in Model Selection |
| --- | --- | --- | --- |
| Bio1 = Annual Mean Temperature | Mean annual air temperature over 1 year | Celsius | Yes |
| Bio2 = Mean Diurnal Range | mean diurnal range of temperatures averaged over 1 year | Celsius | Yes |
| Bio3 = Isothermality | ratio of diurnal variation to annual variation in temperatures | Celsius | No |
| Bio4 = Temperature Seasonality | standard deviation of the monthly mean temperatures | Celsius | Yes |
| Bio5 = Max Temperature of Warmest Month | The highest temperature of any monthly daily mean maximum temperature | Celsius | Yes |
| Bio6 = Min Temperature of Coldest Month | The lowest temperature of any monthly daily mean maximum temperature | Celsius | No |
| Bio7 = Temperature Annual Range | The difference between the Maximum Temperature of Warmest month and the Minimum Temperature of Coldest month | Celsius | Yes |
| Bio8 = Mean Temperature of Wettest Quarter | The wettest quarter of the year is determined (to the nearest month) | Celsius | No |
| Bio9 = Mean Temperature of Driest Quarter | The driest quarter of the year is determined (to the nearest month) | Celsius | No |
| Bio10 = Mean Temperature of Warmest    Quarter | The warmest quarter of the year is determined (to the nearest month) | Celsius | No |
| Bio11 = Mean Temperature of Coldest    Quarter | The coldest quarter of the year is determined (to the nearest month) | Celsius | No |
| Bio12 = Annual Precipitation | Accumulated precipitation amount over 1 year | kg/m^2^ | Yes |
| Bio13 = Precipitation of Wettest Month | The precipitation of the wettest month. | kg/m^2^ | Yes |
| Bio14 = Precipitation of Driest Month | The precipitation of the driest month. | kg/m^2^ | Yes |
| Bio15 = Precipitation Seasonality | The Coefficient of Variation is the standard deviation of the monthly precipitation estimates expressed as a percentage of the mean of those estimates (i.e. the annual mean) | kg/m^2^ | Yes |
| Bio16 = Precipitation of Wettest Quarter | The wettest quarter of the year is determined (to the nearest month) | kg/m^2^ | No |
| Bio17 = Precipitation of Driest Quarter | The driest quarter of the year is determined (to the nearest month) | kg/m^2^ | No |
| Bio18 = Precipitation of Warmest Quarter | The warmest quarter of the year is determined (to the nearest month) | kg/m^2^ | No |
| Bio19 = Precipitation of Coldest Quarter | The coldest quarter of the year is determined (to the nearest month) | kg/m^2^ | No |
